# Single-molecular real-time deep sequencing reveals the dynamics of multi-drug resistant haplotypes and structural variations in the hepatitis C virus genome

**DOI:** 10.1038/s41598-020-59397-2

**Published:** 2020-02-14

**Authors:** Taiki Yamashita, Haruhiko Takeda, Atsushi Takai, Soichi Arasawa, Fumiyasu Nakamura, Yoichi Mashimo, Miyuki Hozan, Shigeru Ohtsuru, Hiroshi Seno, Yoshihide Ueda, Akihiro Sekine

**Affiliations:** 1 Department of Omics-based Medicine, Center for Preventive Medical Sciences, Chiba University, Chiba, Japan; 2 Department of Gastroenterology and Hepatology, Graduate School of Medicine, Kyoto University, Kyoto, Japan; 3 Department of Public Health, Graduate School of Medicine, Chiba University, Chiba, Japan; 4 Department of Genome Medical Support, National Cerebral and Cardiovascular Center, Osaka, Japan; 5 Department of Primary Care and Emergency Medicine, Graduate School of Medicine, Kyoto University, Kyoto, Japan; 6 Department of Gastroenterology and Hepatology, Graduate School of Medicine, Kobe University, Kobe, Japan

**Keywords:** Genomics, Hepatitis C, Hepatitis C virus, Cancer

## Abstract

While direct-acting antivirals (DAAs) for hepatitis C virus (HCV) have dramatically progressed, patients still suffer from treatment failures. For the radical eradication of HCV, a deeper understanding of multiple resistance-associated substitutions (RASs) at the single-clone level is essential. To understand HCV *quasispecies* and their dynamics during DAA treatment, we applied single-molecule real-time (SMRT) deep sequencing on sera from 12 patients with genotype-1b HCV infections with DAA treatment failures, both pre- and post-treatment. We identified >3.2 kbp sequences between NS3 and NS5A genes of 187,539 clones in total, classifying into haplotype codes based on the linkage of seven RAS loci. The number of haplotype codes during the treatment, per sample, significantly decreased from 14.67 ± 9.12 to 6.58 ± 7.1, while the number of nonsynonymous codons on the seven RAS loci, per clone, significantly increased from 1.50 ± 0.92 to 3.64 ± 0.75. In five cases, the minority multi-drug resistant haplotypes at pre-treatment were identical to the major haplotypes at relapse. Moreover, various structural variations (SVs) were detected and their dynamics analysed. These results suggest that SMRT deep sequencing is useful for detecting minority haplotypes and SVs, and to evaluate the dynamics of viral genomes at the single-clone level.

## Introduction

The hepatitis C virus (HCV) has approximately 9.6 kb of a single-stranded RNA genome. After the approval of oral direct-acting antivirals (DAAs), after drastic HCV treatment, levels of HCV-RNA remain undetectable (sustained virological response; SVR) in most patients chronically infected by HCV or suffering from HCV-related diseases^1–5^. In some patients, however, DAAs cannot completely eradicate HCV^1–4^.

One of the major causes of HCV survival during DAA treatment is thought to be a mutation of its genome. Mutations are likely to occur in the HCV genome due to the fact that RNA-dependent RNA polymerase lacks a proofreading function. Therefore, during HCV infection, the population of HCV includes similar but slightly different clones, and HCV is therefore known as a *quasispecies*^6,7^. Some *quasispecies* have resistance-associated substitutions (RASs) and make the DAAs ineffective. For example, Y93H on the NS5A gene is associated with the resistance of NS5A inhibitors^8–11^, while the inframe deletion of the NS5A-P32 codon leads to the failure of glecaprevir and pibrentasvir treatments^12–15^. Besides these resistance mutations, Q80/D168 on the NS3 gene and R30/L31/Q54 on the NS5A gene are associated with RASs in HCV^16,17^. The antiviral treatment of patients after liver transplantation, or of those with liver cirrhosis, is still challenging, and to achieve SVR in some of these cases, drug-resistant HCV must be overcome^3,18^. Thus, a deeper understanding of multi-drug resistant HCV clones and their genetic landscape is important for the radical eradication of HCV in all cases.

To date, Sanger sequencing and second-generation sequencing, such as Miseq/Hiseq (Illumina, San Diego, USA) and Roche 454 sequencing (Roche 454 Life Sciences, Branford, CT, USA), have traditionally been used to determine genome variants^19,20^. However, these sequencers have the following limitations: (1) although these sequencing technologies output accurate sequences, Sanger sequencing cannot distinguish rare variants from noise^21^; (2) second-generation sequencers produce reads that are too short (paired-end 2 × 300 bp reads by Miseq/Hiseq and single-end 700 bp reads by Roche 454 sequencing) to determine multiple RASs from NS3 and NS5A genes (3.2 kbp) on the same genomic regions (linkage); (3) neither sequencer can detect large structural variations (SVs). In contrast, to detect multiple RASs and SVs at a single viral clone level of resolution, deep sequencing using third-generation long-read sequencers, including the PacBio RS II/Sequel (Pacific Biosciences, Menlo Park, CA, USA) and the Nanopore sequencer (Oxford Nanopore Technologies, Oxford, United Kingdom), is considered necessary. These sequencing technologies generate reads of over 10 kbp^22,23^. While the accuracy of the raw sequence reads from third-generation sequencers is limited to 90%, a characteristic error correction methodology by the PacBio sequencer system, called circular consensus sequencing (CCS) technology, improves the accuracy of the raw reads to as high as 99.9%^24^. Due to these characteristics, the PacBio RS II/Sequel can produce high quality sequence reads of HCV genomes at the single-molecule level, including multiple RASs, rare RASs, and SVs.

Recently, we reported a methodology to evaluate viral *quasispecies* using the PacBio RSII sequencer^25^. We analysed the linkage of several RASs of NS3 and NS5A together, with a number of synonymous substitutions at the single viral clone level. Through phylogenetic analysis using the genetic information of hundreds of viral clones in each serum, we demonstrated that multi-drug resistant viral clones could arise from pre-existing minority populations of drug-resistant variants. Although these analytical methods could be useful in unveiling the genetic basis of the evolution of multi-drug resistant viral clones, there is a need to simplify PacBio sequencing output to provide usable and clinically useful information.

Therefore, in this study, we aimed to establish an analytical method to convert the sequencing data from long-read deep sequencing into lower-dimensional haplotype data, using the linkage of seven RAS-related loci and to analyse the data from the numerous viral haplotypes in each sample. Taking advantage of the long-read sequencer, we also explored SVs in each of the HCV genome sequences. Furthermore, using the sequence data at the single viral clone level, we examined the dynamics of the RAS-based haplotypes and the SVs pre- and post-treatment with DAA in treatment failures.

## Results

### Single-molecule real-time sequencing of the region from the NS3 to the NS5A genes in the HCV genome

**Figure 1:**
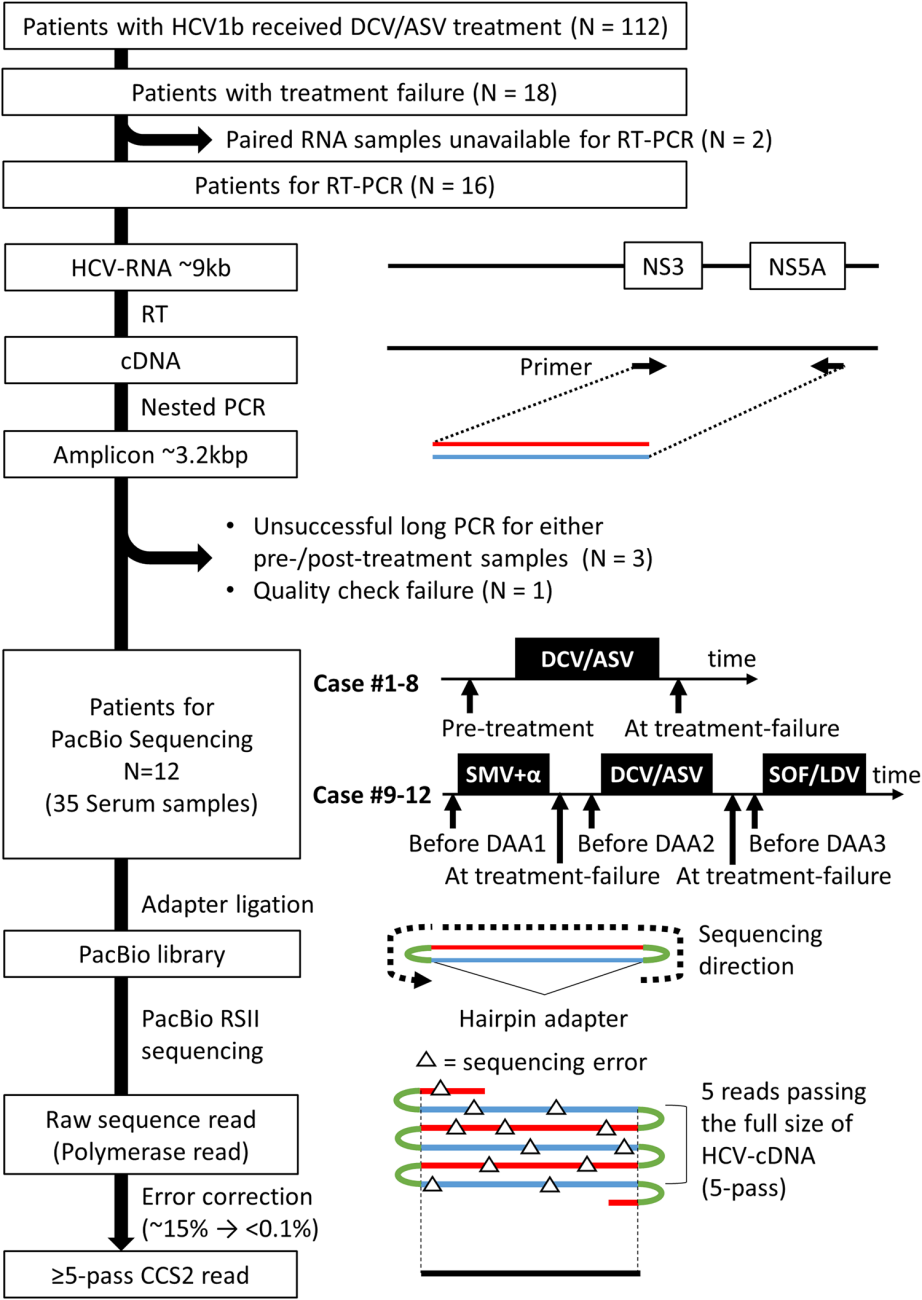
Sample collection and single-molecule real-time sequencing workflow. This flow consisted of sample collection, reverse transcription polymerase chain reaction (RT-PCR), single-molecule real-time (SMRT) sequencing, and Circular Consensus Sequencing2 (CCS2). For cases #1–#8, the samples were extracted prior to treatment and after treatment failure of the daclatasvir + asunaprevir (DCV/ASV). For cases #9–#12, the samples were extracted prior to treatment with simeprevir (SMV) combined with peginterferon and ribavirin, DCV/ASV and sofosbuvir + ledipasvir (SOF/LDV), and after the treatment failure of the SMV and DCV/ASV. RT-PCR amplified the NS3-NS5A gene regions of the cDNA transcribed from HCV-RNA. For SMRT sequencing, we constructed the library by binding the adapters to the cDNA by ligation. The library was sequenced with PacBio RS II. While the sequenced raw reads included sequencing errors, CCS2, the error correction algorithm, generated highly accurate reads by correcting the errors with the raw reads repetitively sequenced in single the molecules.

We conducted SMRT sequencing for the 35 HCV-samples and obtained a total of 9,342,760 raw reads with 25,251,529,303 bp (Fig. 1, Supplementary Table S1). To obtain more accurate reads, we executed a Consensus Circular Sequencing 2 (CCS2) algorithm on the error-prone raw reads using the pbsmrtpipe software (Pacific Biosciences). The CCS2 algorithm interlaces all raw reads (called 1-pass when only one raw read was sequenced from one adapter to another adapter, and called 2-pass when two raw reads were sequenced from one adapter to another adapter) derived from the same DNA template. The pbsmrtpipe and blastn software, and the in-house perl script generated a total of 284,565 ≥ 5-pass CCS2 reads with primer sequences at each end (888,923,070 bp). The CCS2 reads were equivalent to a 7,516× coverage, on average, of the region between the NS3 and NS5A genes (Supplementary Fig. S1). The mean quality value of the ≥5 pass-CCS2 reads was high, at 55.4–73.93, and the sequencing error rate was low, at 2.88 × 10^−6^–4.05 × 10^−8%^.

To assess the error rate in the whole procedure, we used two samples of HCV-containing plasmid as the control sequence. SMRT sequencing generated a total of 819,416 raw reads of the NS3-to-NS5A region with 2,177,357,653 bp. After error correction by the CCS2 algorithm, we obtained 38,364 ≥5-pass CCS2 reads (101,683,536 bp). These ≥5-pass CCS2 reads were aligned to the HCV1b reference (accession no. AB047639.1) using blastn version 2.2.29 with customised parameters (-dust no)^26^. Based on the blastn results, we found the mismatch rate to be 0.023% (SD = 0.039, 95% CI: 0.023–0.024) and the error rate to be 0.267% (SD = 0.298, 95% CI: 0.264–0.270) (Supplementary Table S2).

The sequence data suggested that we obtained more than 7,000 accurate long-reads per sample, derived from the region between the NS3 and NS5A genes.

### Determination of RAS haplotypes of the HCV genome

**Figure 2:**
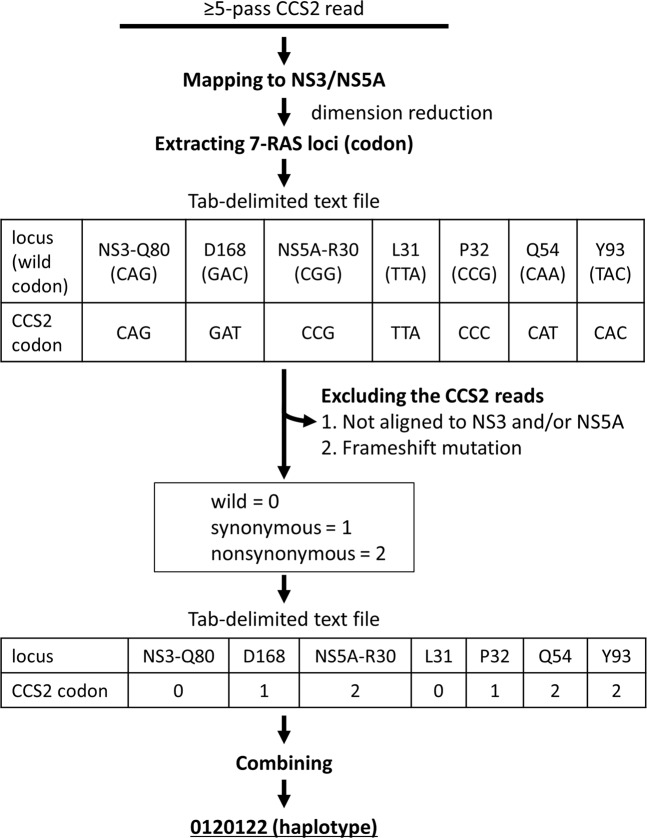
The flow of determining haplotype codes. The method for determining the haplotype code consists of 4 steps: (1) mapping CCS2 reads to the NS3/NS5A gene sequences using blastn, (2) extracting each codon from the 7 RAV-sites in the CCS2 reads, based on the blastn results, converting each codon of the 7 RAV-sites to three numbers (0, 1, and 2), and combining all codons to make a haplotype. The figure shows the example of the steps, from aligning the CCS2 reads to constructing a haplotype.

To analyse the dynamics of the drug-resistant viral clones during the DAA treatment in a simple manner, we determined the linkage of seven RASs (Q80 and D168 in NS3, and R30, L31, P32, Q54, and Y93 in NS5A) from the NS3 to NS5A genes of each viral clone, and reduced the dimensions of the haplotype data (Fig. 2).

First, to determine the position of the NS3 and NS5A genes in each clone, we aligned the ≥5-pass CCS reads to the sequences of the NS3 (3,408–5,300) and NS5A genes (6,246–6,735) in the HCV reference genome (accession no. D90208.1) by blastn version 2.2.29 with customised parameters (-dust no)^26^. According to the results from the blastn, each codon from the CCS2 reads that was in one of the seven RASs, was extracted. Of the CCS2 reads, those possessing frameshift indels or not mapped to NS3 or NS5A were excluded from haplotype determination. Next, to convert the linkage information of the seven codon changes to low-dimensional digital data, we encoded the original codon (wild codon) as 0, a synonymous codon as 1, and a nonsynonymous codon as 2 (Fig. 2). The original codons and the synonymous codons for the seven-RAS loci are defined in Supplementary Table S3. After translating each codon to the code, we constructed a 7-digit code for 7-RAS haplotypes by combining these codes (Fig. 2). Before applying the coding procedure, the total number of HCV clones between the NS3-NS5A genes was 187,539, and the average number was as high as 5,358 (SD = 3460.05) haplotypes per sample (Table 1). In contrast, after the construction of the 7-digit coded haplotypes, the number of the viral haplotypes were summarised to 24 on average (SD = 20.45). Consequently, the coding procedure simplified thousands of HCV clone types into fewer than one hundred types, using the haplotype code for the 7-RAS loci, making the overall picture of the HCV *quasispecies* in each sample clearer and easier to understand.

**Table 1:** Sample information.

Case	Sample	Timepoint	Description	HCV RNA^a^	serum ALT^b^	# of HCV clones^c^	# of haplotype codes
#1	#1-DCV/ASV-pre	1	before DCV/ASV	6.6	43	337	6
	#1-DCV/ASV-post	2	after DCV/ASV	5.2	28	6,949	13
#2	#2-DCV/ASV-pre	1	before DCV/ASV	6.6	33	8,599	52
	#2-DCV/ASV-post	2	after DCV/ASV	5	32	257	5
#3	#3-DCV/ASV-pre	1	before DCV/ASV	5.5	29	5,583	23
	#3-DCV/ASV-post	2	after DCV/ASV	4.8	26	142	5
#4	#4-DCV/ASV-pre	1	before DCV/ASV	5.4	42	1,469	21
	#4-DCV/ASV-post	2	after DCV/ASV	5.2	25	12,525	11
#5	#5-DCV/ASV-pre	1	before DCV/ASV	7.1	13	10,854	32
	#5-DCV/ASV-post	2	after DCV/ASV	6.2	14	7,743	12
#6	#6-DCV/ASV-pre	1	before DCV/ASV	6.1	30	1,850	26
	#6-DCV/ASV-post	2	after DCV/ASV	5.8	24	4,117	8
#7	#7-DCV/ASV-pre	1	before DCV/ASV	6.2	65	2,273	49
	#7-DCV/ASV-post	2	after DCV/ASV	5.5	31	5,429	11
#8	#8-DCV/ASV-pre	1	before DCV/ASV	6.3	81	3,954	39
	#8-DCV/ASV-post	2	after DCV/ASV	3.8	20	340	6
#9	#9-SMV-pre	1	before SMV/PegIFN/RBV	7.8	119	6,257	15
	#9-SMV-post	2	after SMV/PegIFN/RBV	4.5	9	3,678	10
	#9-DCV/ASV-pre	3	before DCV/ASV	7.2	76	5,454	35
	#9-DCV/ASV-post	4	after DCV/ASV	4.7	21	9,917	12
	#9-SOF/LDV-pre	5	before SOF/LDV	7.8<	136	8,556	24
#10	#10-SMV-pre	1	before SMV/PegIFN/RBV	6.8	55	6,923	17
	#10-SMV-post	2	after SMV/PegIFN/RBV	6.6	28	7,065	18
	#10-DCV/ASV-pre	3	before DCV/ASV	6.2	81	7,184	13
	#10-DCV/ASV-post	4	after DCV/ASV	3.5	16	9,942	12
	#10-SOF/LDV-pre	5	before SOF/LDV	5.9	52	11,858	12
#11	#11-SMV-pre	1	before SMV/PegIFN/RBV	6.9	66	2,763	10
	#11-DCV/ASV-pre	2	before DCV/ASV	5.7	18	6,285	15
	#11-DCV/ASV-post	3	after DCV/ASV	7.2	89	1,645	6
	#11-SOF/LDV-pre	4	before SOF/LDV	6.3	66	1,499	53
#12	#12-SMV-pre	1	before SMV/PegIFN/RBV	7.6	38	7,235	25
	#12-SMV-post	2	after SMV/PegIFN/RBV	7.6	31	7,273	73
	#12-DCV/ASV-pre	3	before DCV/ASV	7.2	50	6,506	92
	#12-DCV/ASV-post	4	after DCV/ASV	7.4	17	628	30
	#12-SOF/LDV-pre	5	before SOF/LDV	7	31	4,450	58

DCV, daclatasvir; ASV, asunaprevir; SMV, simeprevir; PegIFN, peginterferon; RBV, ribavirin; SOF, sofosbuvir; LDV, ledipasvir; ^a^logIU/mL; ^b^U/L; ^c^The number of CCS2 reads except for the same sequences as other reads in comparing CCS2 reads in sample each other by blastn.

### Dynamics of the 7-RAS haplotypes between the DAA pre- and post-treatment

**Figure 3:**
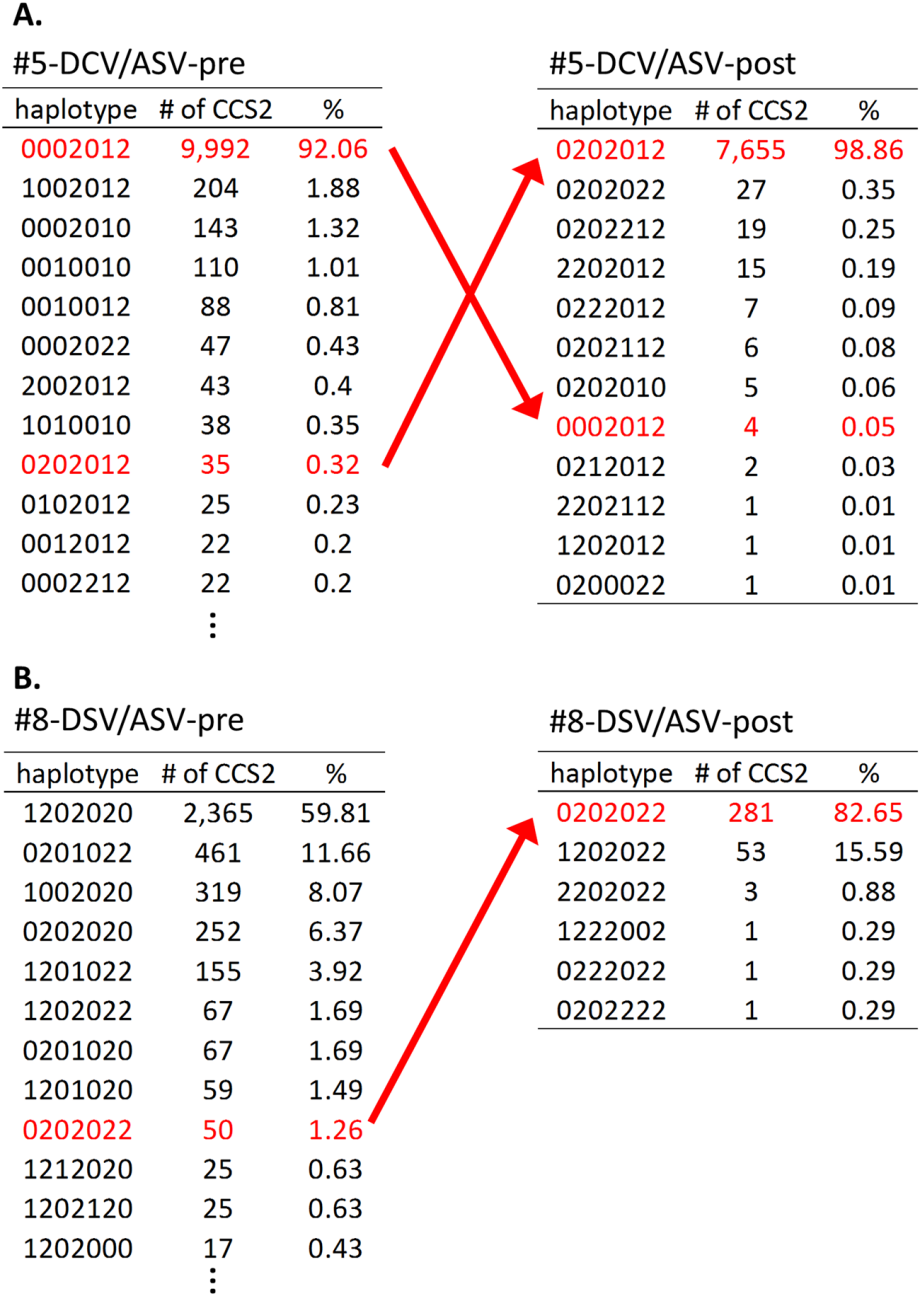
Dynamics of haplotype codes before treatment and after treatment failure. (**A**) Comparison of the haplotypes between #5-DCV/ASV-pre and #5-DCV/ASV-post. Before therapy, “0202012”, resistant to both the NS3 inhibitor and NS5A inhibitor, existed at a frequency of 0.32% (left table). In contrast, the relative abundance of “0202012” increased to 98.86%, with treatment failure (right table). The % in each table represents the percentage for the number of the CCS2 reads in the sample. (**B**) Comparison of haplotypes between #8-DCV/ASV-pre and #8-DCV/ASV-post. Although haplotype “0202022” was 1.26% and the minor haplotype at pre-treatment, this haplotype was 82.65%, and the major haplotype at treatment failure. The % in each table represents the percentage for the number of the CCS2 reads in the sample.

To examine the dynamics of the 7-RAS haplotypes, we listed the haplotype codes at each timepoint and compared the relative frequency of the 7-RAS haplotypes between the pre- and post-treatment samples of a total of 15 DAA treatments (before and after DCV/ASV in cases #1–#12, and before and after SMV in cases #9, #10 and #12; Supplementary Table S4). Then, to exclude the haplotypes derived from artefacts, we set the mismatch rate + 2 SD as the threshold (0.103%) and listed the haplotype codes with over 0.103% frequency in the samples. In 5 of the 15 paired comparisons (#5-DCV/ASV-pre/post, #8-DCV/ASV-pre/post, #9-SMV-pre/post, #10-SMV-pre/post and #10-DCV/ASV-pre/post), the major haplotypes had multiple RASs present when the treatment failed, also existing in 0.14–1.26% of the CCS2 reads at pre-treatment. Meanwhile, in the other ten paired comparisons, the major haplotypes only had RASs present when the treatment failed. Of these 5 paired comparisons, the dynamics of the haplotypes in the 2 representative cases are demonstrated in Fig. 3 (#5-DSV/ASV-pre/post paired samples and #8-DSV/ASV-pre/post paired samples).

During the DCV/ASV treatment in case #5 (Fig. 3A), the haplotype code “0202012” was the ninth most frequent haplotypes in the pre-treatment samples. However, after treatment, haplotype “0202012” increased to the most frequent haplotype. Likewise, with the example of case #5 (Fig. 3B), its ninth most frequent haplotype before treatment, “0202022”, became the most frequent haplotype after treatment failure, and showed resistance to DCV and ASV due to the nonsynonymous changes of NS3-D168, NS5A-L31, and NS5A-Y93. These data suggest that low-abundance multidrug-resistant viral clones exist before the DAA treatment.

**Figure 4:**
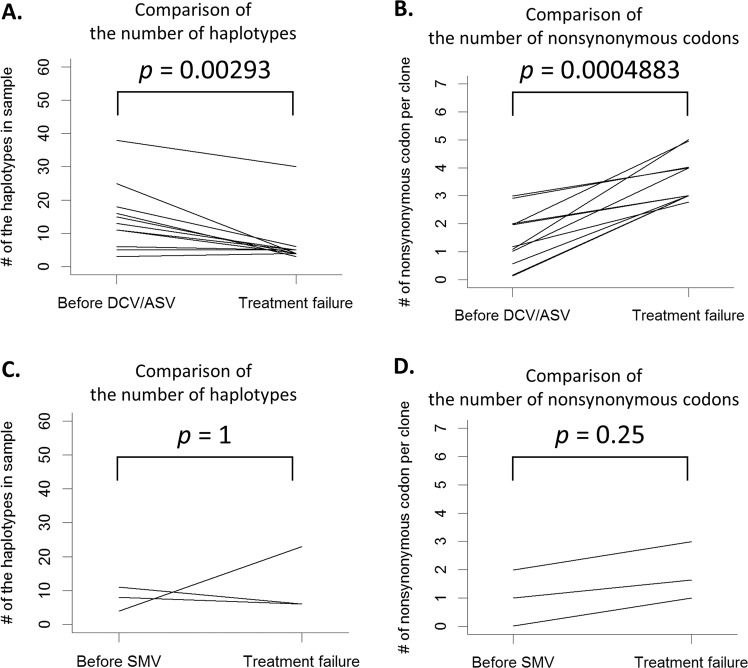
Differences in the haplotype profiles, both pre- and post-treatment on daclatasvir/asunaprevir and simeprevir. **(A)** The graph demonstrates the differences in the number of haplotype codes between pre-and post-treatment with daclatasvir/asunaprevir (DCV/ASV). The x-axis represents the timepoint of the sera collection, and the y-axis represents the number of haplotypes. The Wilcoxon signed-rank test was used for the statistical examination of the difference between the number of haplotype codes. **(B)** The graph demonstrates the difference in the number of nonsynonymous codons per clone from pre-treatment of DCV/ASV to post-treatment. The x-axis represents the timepoint of the sera collection and the y-axis represents the mean number of nonsynonymous codons on the 7-RAS loci, per HCV clone. The Wilcoxon signed-rank test was used for the statistical examination of the differences between the number of nonsynonymous codons. **(C)** The graph demonstrates the differences in the number of haplotype codes between pre-and post-treatment with simeprevir (DCV/ASV). The x-axis represents the timepoint of the sera collection, and the y-axis represents the number of haplotypes. **(D)** The graph demonstrates the difference in the number of nonsynonymous codons per clone from the pre-treatment of SMV to the post-treatment. The x-axis represents the timepoint of the sera collection and the y-axis represents the mean number of nonsynonymous codons on the 7 RAS loci, per HCV clone.

We also focused on the change in the number of haplotype codes from the pre-treatment of DAAs (DCV/ASV and SMV) to relapse (Fig. 4A). With DCV/ASV treatment, the number of haplotypes significantly decreased by 8.08 (SD = 18.77, 95% CI: 4.5–12.5) from 14.67 (SD = 9.12) with pre-treatment of DCV/ASV, and to 6.58 (SD = 7.1) at relapse (*p* = 0.00293). In contrast, comparing the means of the nonsynonymous codons of the 7-RAS loci per CCS2 read before and after DSV/ASV therapy, the number of nonsynonymous codons significantly increased by 2.14 (SD = 0.96, 95% CI: 1.35–2.91) from 1.50 (SD = 0.92) at the pre-treatment to 3.64 (SD = 0.75) at treatment failure (p = 0.0004883) (Fig. 4B). With SMV treatment, neither the number of haplotype codes (mean = 4, SD = 10.68, 95% CI: −5–19) nor the number of nonsynonymous codons (mean = 0.87, SD = 0.17, 95% CI: 0.63–1.00) showed significant differences, due to the small paired-sample size (Fig. 4C,D).

Although the coded haplotypes lacked the detailed information of each codon, the comparison of haplotype codes during DAA therapy suggests that rare HCV clones with multiple RASs at pre-treatment might be the cause of relapse as they became the major haplotype after treatment failure. In addition, the significant reduction in the number of haplotype codes and the significant gain in the nonsynonymous codons at RAS loci at treatment failure, indicates the clonal selection of viruses with survival benefit under anti-HCV treatment.

### Structural variations detected by SMRT sequencing

**Figure 5:**
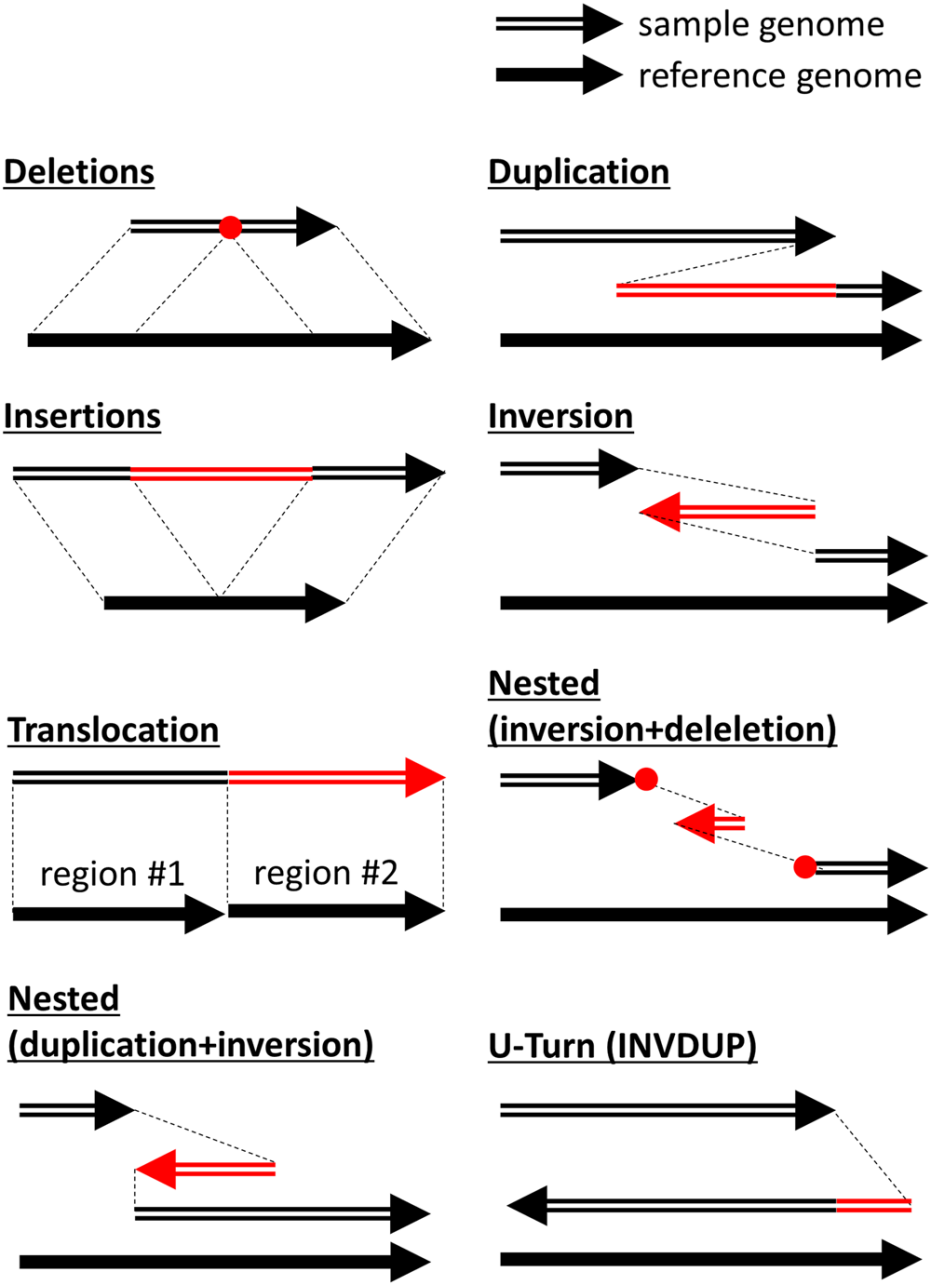
The definition of structural variations (SVs) by Sniffles. The figure is adapted from the article of Sniffles^32^ with focus on the relationship between the reference genome sequence and the sample genome sequence with each SV^32^. Sniffles is able to detect 8 types of SVs, such as deletions, duplications, insertions, inversion, translocation, nested variation (inversion + deletion and duplication + inversion), and U-Turns (INVDUP). The red regions indicate regions where an SV occurs. The red points stand for the breakpoint of deletions.

To understand the viral clones at a deeper level, we analysed structural variation in the HCV-RNA genome at single-clone resolution. To call candidates with ≥30-bp SVs, we executed ngmlr and Sniffles for each sample. In all cases, a total of 6,512 CCS2 reads had SVs, corresponding to 2.29% of all ≥- 5-pass CCS2 reads. In particular, deletions were detected in 4,393 of CCS2 reads (1.54%), while duplications, insertions, inversions, and U-turns (INVDUP) were detected in 220 (0.08%), 61 (0.02%), 68 (0.02%), and 1,906 reads (0.67%), respectively (Fig. 5, Supplementary Table S5).

**Figure 6:**
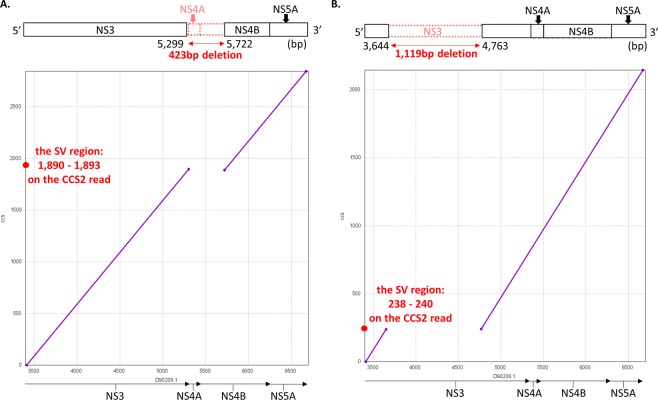
The detection of the genome structure harbouring SVs between the NS3 and NS5A gene regions. The mapping software ngmlr version 0.2.6 aligned ≥5-pass CCS reads to the HCV genome, and the structural variation (SV)-calling software, Sniffles, detected ≥30-bp SVs. The figure demonstrates that two deletions exist at a relative abundance of ≥1% in the samples. In each panel, the upper schema shows the HCV genome with SV from the NS3 to the NS5A gene regions. The red symbols stand for the region that the SV in question occurred in. In particular, the inverted letters in the schema mean “inversion,” and the boxes with dash lines mean “deletion.” The genome positions described here are based on the HCV reference sequence (accession no. D90208.1). The lower image demonstrates the alignment of the representative reads supporting the SVs by the MUMmer program. The x-axis shows the position of the HCV reference genome. The Y-axis shows the position of the reads harbouring SVs. The purple solid line shows the alignment for an HCV reference genome without SVs. The blue solid line shows the inverted alignment for an HCV reference genome. The red point shows the SV region on the CCS2 read, which was used for SV validation. **(A)** In sample #12-SMV-post, a 423-bp deletion was detected. **(B)** In #12-SOF/LDV-pre, 1,119-bp deletion was clonally detected.

We focused on the 10 SVs detected with frequencies of ≥1% of the CCS2 reads, and examined the changes in their frequencies after the treatment (Supplementary Fig. S2, Supplementary Table S6). Two examples (SV7 and 10) of the 10 SVs are shown in Fig. 6A,B, respectively. In the case of SV7, the CCS2 reads lacked the sequence located at the region between 5,299–5,722 on the HCV reference genome (accession no. D90208.1), including parts of NS3, NS4A, and NS4B (Fig. 6A). In the validation, two of the Ion Proton reads were aligned to the region the SV occurred in. Also, in the case of SV10 (Fig. 6B), the CCS2 reads lacked the sequence between the 3,644–4,763, including part of NS3. In the validation, 925 of the Ion Proton reads were aligned to the region the SV occurred in. Of these 10 SVs, six (SV1, 2, 4, 5, 6, and 8) existed at the pre-treatment and vanished after the treatment. In contrast, two SVs (SV3 and 7) appeared for the first time after treatment failure. The remaining SV (SV9) was detected throughout the treatment period.

As shown in these SVs, we identified HCV clones harbouring simple SVs and their various dynamics.

## Discussion

To completely eradicate HCV, a deep understanding of the genetic background behind multidrug-resistant HCV clones is important. However, as second-generation sequencers generate up to 2 × 300  p paired-end reads, deep sequencing with these technologies cannot detect the linkage of multiple RASs between NS3 and NS5B genes and elucidate multi-drug resistance at single clone level. In the current study, we have established a methodology to evaluate viral *quasispecies* by linkage information of multiple RASs from NS3 to NS5A genes, on every viral clone in the sera, using recently developed SMRT sequencing technologies, which can generate extremely long sequence reads.

We have previously applied SMRT sequencing to evaluate viral *quasispecies* and the generation process of multi-drug resistant viral clones at the single viral clone level^25^. The amount of sequencing data, however, was extremely large, and the methodology required to conduct the subsequent phylogenetic analysis extremely complicated, especially when considering a common use-case of this technology: as a part of bed-side examinations in a clinical setting. In contrast, in the current study, we converted the complicated information of multiple codon changes during treatment into a simple seven-digit code, associated with drug resistance in each sequence read. Using this simplified methodology, the large quantity of data from the SMRT sequencing was aggregated into a simpler form that enabled easier evaluation of the complicated viral heterogeneity of HCV and its dynamics during DAA treatment.

Another advantage of the current study is the establishment of long-read deep sequencing for a viral genome, using the CCS2 algorithm for error correction. In our previous report on HCV analysis using SMRT sequencing, we used 10-pass CCS reads to evaluate only accurate sequence reads, resulting in approximately 200–500 clones, with 99.65% accuracy per sample, eligible for analysis. These methods could miss rare variants in each serum sample. To improve this, in the current study, we used CCS2 reads generated by the new error correction algorithms, which are more accurate than the previously used CCS reads, and a total of 187,539 CCS2 reads in the 35 samples could be analysed with 99.73% accuracy. The 5,358 HCV clones found per sample indicate that mutations constantly occur in the HCV genome, resulting in the amplification of extremely diverse HCV clones *in vivo*. In addition, comparing the long-read deep sequencing data between the pre- and post-treatments, we found that the major 7-RAS haplotypes with multi-drug resistance in post-treatment existed at low abundance during pre-treatment. This highlights how our strategy, with long-read deep sequencing and encoding of viral haplotypes, could be utilised to analyse the dynamics of multi-drug resistant viral clones.

Taking advantage of long-read sequencing methodology, we also identified several SVs including long deletions or U-turns (INVDUP) on the HCV RNA genome and compared the frequencies of the SVs during the treatments. In addition, to the best of our knowledge, this study is the first investigation of the dynamics of HCV mutants harbouring SVs during treatment, where dynamics include the appearance and disappearance of HCVs at relapse. Among the SVs reported in the HCV genome, defective HCV clones lacking a subset of structural proteins are occasionally detected in some patients’ sera^27^. We should consider that reports of defective HCV arise from the potential artefacts generated during the RT-PCR process. However, if the defective HCV indeed exists in patients’ sera, they may be amplified in the patients’ hepatocytes, not by their NS proteins but rather using the proteins of co-infecting full-length HCV clones. It remains unclear how these incomplete genomes are formed and maintained and whether they are associated with drug resistance or the escape from host immunity. In addition, recombinant HCV clones with different genotypes have also been reported^28^, although it is not clear how these viral clones were generated.

One of the limitations of our study is its small sample size. We analysed only three paired samples of SMV treatment and did not study the dynamics of 7-RAS haplotypes during SMV treatment. Also, although we evaluated the dynamics of the 7-RAS haplotypes in HCV samples, we could not find how the distribution of these haplotypes influences HCV genome replication and treatment outcome. To investigate the relationship between HCV *quasispecies* and these outcomes, a larger sample size than that of the current study would be required.

The second limitation arises from the elimination of detailed nucleotide information, which might be associated with treatment efficacy, in exchange for simplification of the haplotype code. However, the simple digital codes can be generated from any locus of interest to the investigators. Thus, according to the study design, the essential loci may be included in the digital haplotype code and their dynamics can easily be analysed. Although we suggest that HCV clones with multiple RASs may in some cases become the most frequent clones at treatment failure, the possibility cannot be excluded that selection was due to other mutations than those of the 7-RAS loci used for the generation of the haplotype code. Moreover, for a deeper understanding of their dynamics, NS5B and other genes should be analysed using whole-genome SMRT sequencing.

The last limitation of this study is that we cannot show how SVs and the dynamics described in this study affect the biological features of HCV and affected patients. To elucidate the function of SVs in the HCV genome, further analysis with a greatly increased HCV sample size is required.

In conclusion, we have established an encoding methodology to evaluate the dynamics of drug-resistant 7-RAS haplotypes. Coding each RAS greatly simplified the interpretation and analysis of viral haplotypes. In addition, taking advantage of long-read sequencing, we identified several SVs and described their dynamics in HCV genomes, although their virological significance should be further investigated in the future. Importantly, using our methodology, which simplifies the unwieldy data that is the output of third-generation sequencers into simple digital code data, SMRT sequencing could be applied to large-scale investigations with much larger sample sizes. Moreover, it is ideal to be applied to other viruses like hepatitis B viruses (which cannot be completely eliminated from the host) and human immunodeficiency viruses (whose multi-drug resistant variants are currently a clinically significant issue).

## Materials and Methods

### Patients and sample collection

Patients with genotype 1b HCV infections, who were assigned to receive DCV/ASV therapy at Kyoto University, were enrolled in this study from September 2014 to December 2016 (Fig. 1). This manuscript includes reanalysis of clinical samples and sequence data from a previously published article^25^. Of a total of 112 patients who received DCV/ASV treatment, 18 failed to display SVR. We performed reverse transcription polymerase chain reaction (RT-PCR) on 16 samples, as described below. The samples of two further patients were not available for RT-PCR. Following the RT-PCR procedure, the samples from 12 patients underwent single-molecule real-time (SMRT) sequencing as described in the following section.

As a result, 35 HCV-RNA samples from 12 patients who experienced treatment failure with DAA were analysed in this study. Sixteen samples from eight patients (Cases #1–#8) were paired samples before and after receiving NS5A inhibitor daclatasvir and NS3 protease inhibitor asunaprevir (DCV/ASV) combination therapy (Fig. 1). Nineteen samples from four patients (Cases #9–#12), who received treatment with the NS3 inhibitor simeprevir (SMV) combined with peginterferon and ribavirin therapy, DCV/ASV treatment and the NS5B polymerase inhibitor sofosbuvir and NS5A inhibitor ledipasvir (SOF/LDV) combination therapy, were sequentially collected: (1) before SMV treatment; (2) at relapse after SMV treatment; (3) before DCV/ASV treatment; (3) at relapse after DCV/ASV treatment; and/or (4) before SOF/LDV treatment. All 4 patients (Case #9–#12) finally achieved SVR after SOF/LDV therapy, and thus there were no serum samples to analyse afterwards. The patients’ characteristics and sample information are summarised in Tables 1 and S7.

Total-RNA was extracted from serum using the QIAquick Viral Mini kit (Qiagen, Valencia, CA, USA) following the manufacturer’s protocol. Written informed consent was obtained from each patient prior to DAA therapy. All protocols were approved by the ethics committee of Kyoto University and Chiba University. This study protocol complied with all provisions of the Declaration of Helsinki.

### Single-molecule real-time (SMRT) sequencing

We amplified 3120-bp HCV sequences between the NS3 and NS5A genes by using the PrimeScript One Step RT-PCR kit (Takara Bio, Shiga, Japan) and PrimeSTAR HS kit (Takara) according to the manufacturer’s protocol (Fig. 1, Supplementary Materials). Also, we prepared two samples of HCV-containing plasmids for control experiments and amplified the NS3-to-NS5A region with the above method. The primers for RT-PCR are shown in Supplementary Table S8. The PacBio DNA library was constructed from purified DNA product (5 µg) using a DNA Template Prep Kit 3.0 (Pacific Biosciences, Menlo Park, CA, USA) according to the PacBio standard template prep protocol (Pacific Biosciences)^29^. The DNA library was sequenced using PacBio RS II following the protocol from Pacific Biosciences. We used P6C4 polymerase for the sequencing reaction and 6-h movie windows for signal detection.

### Circular consensus sequencing

To improve the accuracy of the sequenced raw reads, fasta/fastq files of the Circular Consensus Sequence 2 (CCS2) reads were generated using pbsmrtpipe 0.44.8 with CCS2 default settings (Fig. 1). After obtaining the CCS2 reads, we extracted the ≥5-pass CCS2 reads, using perl version 5.18.2. To extract the ≥5-pass CCS2 reads with nested PCR primer sequences, the CCS2 reads were mapped to the primer sequences by blastn version 2.2.29 with specific parameters (-word_size 4 -reward 1 -penalty -3)^26^. Based on the blastn results, we extracted the CCS2 reads aligned to the primer on each end with ≥85% of identity. To examine the coverage of these CCS2 reads, we used BLASR version 1.3.1 for mapping CCS2 reads to the HCV reference genome, and GATK version 3.3.2 for calculating the coverage of the CCS2 reads with parameter “-T DepthOfCoverage -DBQ 0^30,31^”. The sequence data for the HCV clones with novel sequences identified in this study have been submitted to the DDBJ/EMBL/GenBank databases under accession number DRA009132^25^.

### Determination of structural variations

For the detection of the structural variations of the HCV-RNA genome, all ≥5-pass CCS2 reads were aligned to the HCV reference genome sequence (accession no. D90208.1) using ngmlr version 0.2.6 with the default parameters^32^. Based on the data of the mapped sequences, structural variations (SVs) were called by Sniffles version 1.0.11 with customised parameters (-t 36 -s 1 -n -1 -r 500 -d 100–cluster–report_seq–ccs_reads)^32^. Sniffles detected ≥30-bp SVs and defined the types of SVs as described in Fig. 6. We evaluated these SVs according to Sniffles’ vcf/bedpe output. To exclude artefacts, SVs identified in only a single CCS2 read and multiple SVs in a single read were filtered out. For the visualisation of the SVs, we used nucmer (MUMmer4 package) with adjusted parameters “-maxmatch -l 10” and mummerplot (MUMmer4 package) with default parameters^33^.

The detected SVs were validated by the reads generated from Ion Proton Sequencer (ThermoFisher Scientific, Waltham, MA, USA)^25^. We generated the libraries from 100 ng of genomic DNA and an Ion Xpress Plus Fragment Library Kit including the Ion Shear Plus Reagents Kit. The amplicons described in the section of SMRT sequencing were ligated to the Ion Xpress Barcode Adapters. TapeStation with a DNA 1000 kit (Agilent, Santa Clara, CA, USA) was used for the visualisation of the size range. We also used Ion Library Taqman Quantitation Kit to identify the library concentration. Following the Ion PI Template OT2 200 Kit User Guide, the diluted library was utilised as a template for clonal amplification on Ion Sphere particles at the emulsion PCR stage. We performed the sequencing over 400 cycles with the Ion PI Sequencing 200 Kit v3 on an Ion PI Chip (Life Technologies).

We aligned Ion Proton reads to SV regions in the CCS2 reads by blastn version 2.2.29^26^. If the multiple Ion Proton reads were aligned to the SV regions with >95% similarity, we considered that the SV existed in the HCV clones.

### Statistical analysis

To compare the number of haplotypes and the number of nonsynonymous codons per HCV clone between the samples prior to DAA treatment and at treatment failure, continuous variables were analysed using the Wilcoxon signed-rank test. Data were analysed using R ver.3.3.2. Two-tailed probability values of p < 0.05 were considered significant.

## Supplementary information

Supplementary Information.

Supplementary Information2.

## Data Availability

The sequence reads are available in the DNA Data Bank of Japan Sequence Read Archive under accession number DRA009132, [https://trace.ddbj.nig.ac.jp/DRASearch/submission?acc=DRA009132].
