# Supplementary material for: Single-molecular real-time deep sequencing reveals the dynamics of multi-drug resistant haplotypes and structural variations in the hepatitis C virus genome
Source: Sci Rep. 2020 Feb 14;10:2651. doi: 10.1038/s41598-020-59397-2 (PMC7021670; doi:10.1038/s41598-020-59397-2)
Supplement: Supplementary file 1 — Supplementary Information. [file 41598_2020_59397_MOESM1_ESM.pdf]

Supplementary Information

Single-molecular real-time deep sequencing reveals the dynamics of multi-drug resistant haplotypes and structural variations in the hepatitis C virus genome

Taiki Yamashita[1], Haruhiko Takeda[1,2], Atsushi Takai[2], Soichi Arasawa[2], Fumiyasu Nakamura[2], Yoichi Mashimo[3], Miyuki Hozan[4], Shigeru Ohtsuru[2,5], Hiroshi Seno[2], Yoshihide Ueda[2,6] and Akihiro Sekine[1,4,*]

[1] Department of Omics-based Medicine, Center for Preventive Medical Science, Chiba University, Chiba, Japan

[2] Department of Gastroenterology and Hepatology, Graduate School of Medicine, Kyoto University, Kyoto, Japan

[3] Department of Public Health, Graduate School of Medicine, Chiba University, Chiba, Japan

[4] Department of Genome Medical Support, National Cerebral and Cardiovascular Center, Osaka, Japan

[5] Department of Primary Care and Emergency Medicine, Graduate School of Medicine, Kyoto University, Kyoto, Japan

[6] Department of Gastroenterology and Hepatology, Graduate School of Medicine, Kobe University, Kobe, Japan

[*] Correspondence to Akihiro Sekine, Ph.D.

Department of Omics-based Medicine, Center for Preventive Medical Science,

Chiba University, Chiba, Japan,

1-8-1, Inohana, Chuo-ku, Chiba, Japan,

Tel: +81-226-2537.

Email: sekine.akihiro@chiba-u.jp

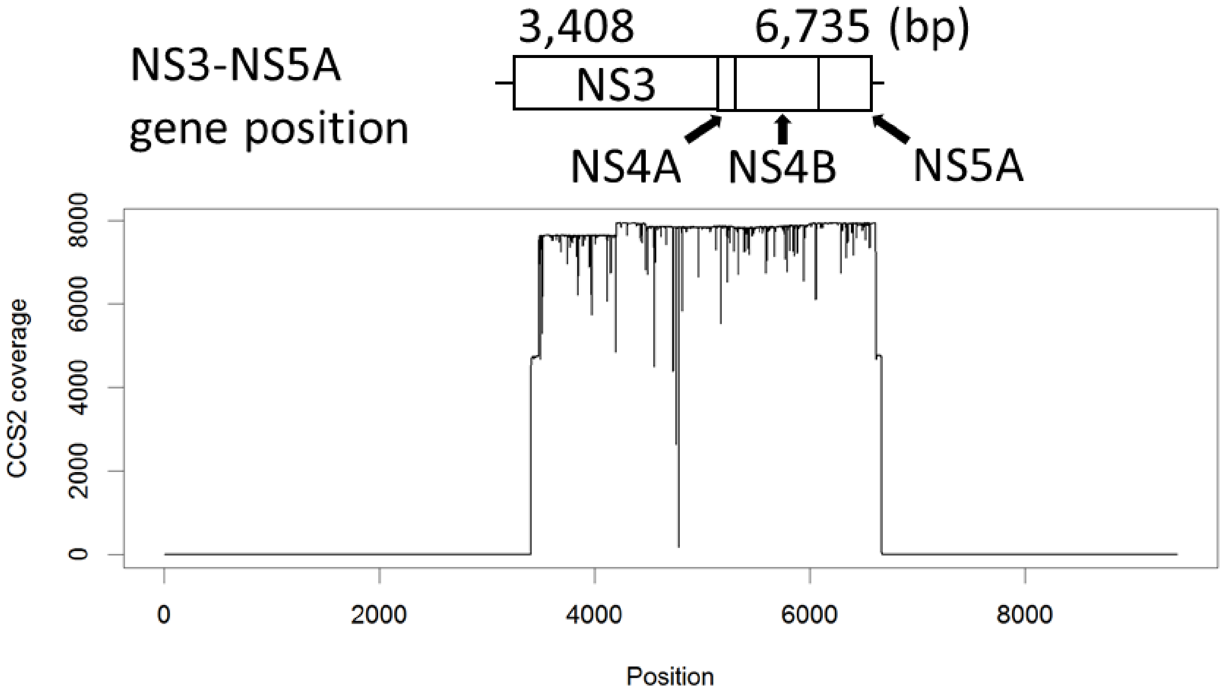

Supplementary Figure S1. Mean coverage of ≥5pass CCS2 reads for HCV genome.

BLASR version 1.3.1 mapped ≥5pass CCS2 reads to HCV genome sequence (accession no. D90208.1) with default parameter and GATK version 3.2.2 calculated the coverage of ≥5pass CCS2 reads with parameter "-T DepthOfCoverage -DBQ 0). X-axis demonstrates the position based on HCV reference genome. Y-axis means the coverage of the CCS2 reads aligned to the position. The CCS2 reads covered the region between NS3 and NS5A genes at >7,000 coverage on average.

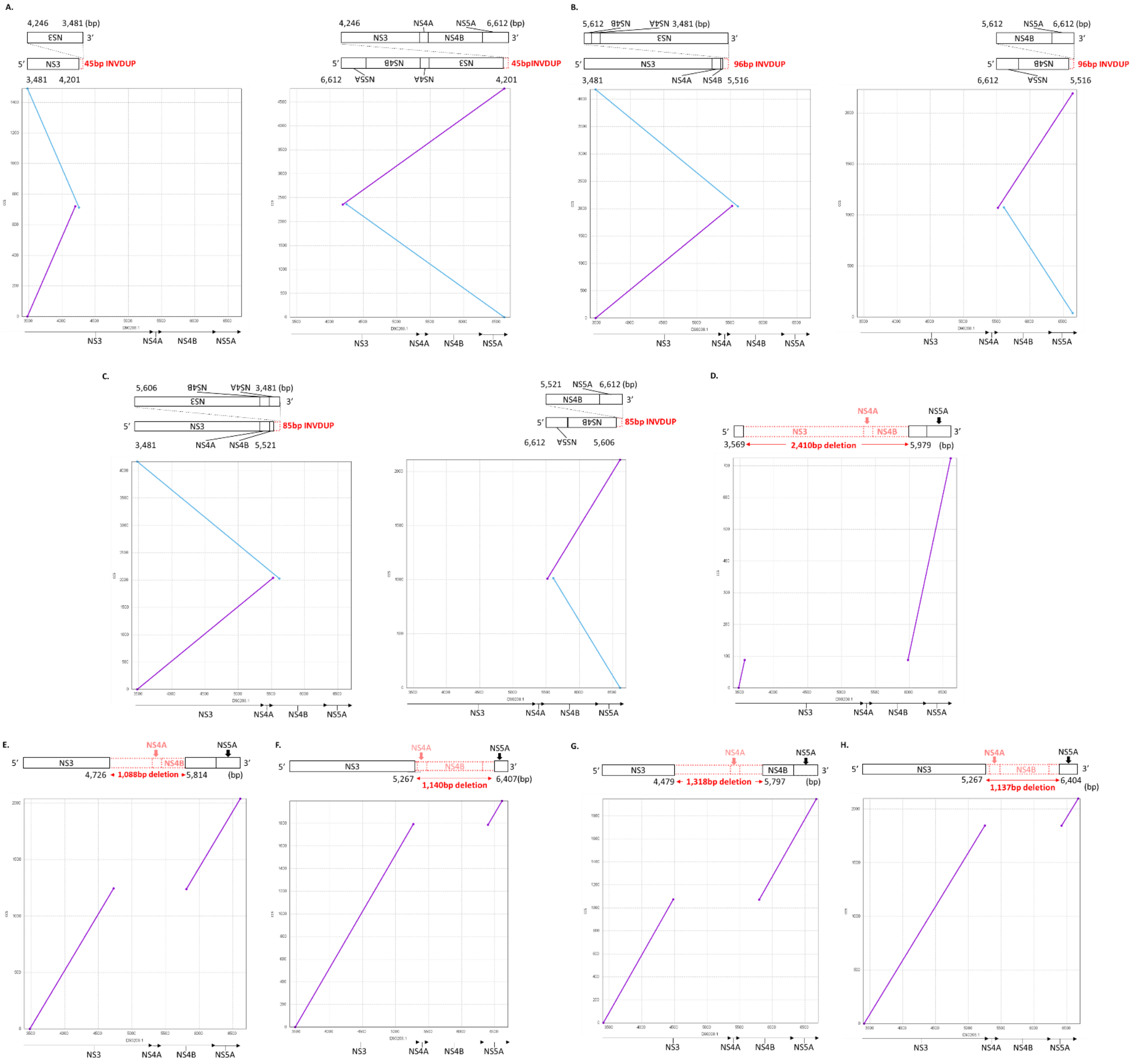
A.
4,246 3,481 (bp)
5′ NS3 3′
45bp INVDUP
3,481 4,201
4,246 NS4A NS5A 6,612 (bp)
NS3 NS4B
45bpINVDUP
6,612 4,201
NS3 NS4A NS4B NS5A
B.
5,612 3,481 (bp)
96bp INVDUP
3,481 NS4A NS4B 5,516
5,612 NS5A 6,612 (bp)
NS4B
6,612 5,516
C.
5,606 3,481 (bp)
NS3
85bp INVDUP
3,481 NS4A NS4B 5,521
5,521 NS5A 6,612 (bp)
NS4B
85bp INVDUP
6,612 5,606
D.
NS4A NS5A
NS3 NS4B
3,569 2,410bp deletion 5,979 (bp)
E.
NS4A NS5A
NS3 NS4B
4,726 1,088bp deletion 5,814 (bp)
F.
NS4A NS5A
NS3 NS4B
5,267 6,407(bp)
1,140bp deletion
G.
NS4A NS5A
NS3 NS4B
4,479 1,318bp deletion 5,797 (bp)
H.
NS4A NS5A
NS3 NS4B
5,267 6,404
1,137bp deletion (bp)

Supplementary Figure S2. The mapping of CCS2 supporting ≥1% SVs to HCV genome.

The mapping software ngmlr version 0.2.6 aligned ≥5-pass CCS reads to HCV genome (accession no. D90208.1), and the structural variation (SV) calling software Sniffles detected ≥30-bp SVs. Of the called SVs, we selected the 10 examples existing at ≥1% in the samples, which were not shown in **Figure 4**. In each panel, the upper schema shows the HCV genome structure with SV at NS3 through NS5A genes. The red symbols stand for the region SV occurred in. Especially, the inverted gene symbols represent inversion occurring in the gene and the boxes with dash lines means deletion. The positions shown in the upper figure are based on the reference. The lower image demonstrates the alignment of the representative reads supporting the SVs in MUMmer program. X-axis shows the position of the HCV reference genome. Y-axis shows the position of the reads harbouring SVs. The purple solid line means the alignment for HCV reference genome without SVs. The blue solid line demonstrates the inverted alignment for HCV reference genome. (A). 45-bp INVDUP within the sample #2-DCV/ASV-pre. (B). 96-bp INVDUP within the sample #4-DCV/ASV-pre. (C). 85-bp INVDUP within #4-DCV/ASV-post. (D). 2 410-bp deletion within #5-DCV/ASV-pre. (E). 1 088-bp deletion within #6-DCV/ASV-pre. (F). 1 140-bp deletion within ##8-DCV/ASV-pre. (G). 1 318-bp deletion within #12-DCV/ASV-pre. (H). 1 137-bp deletion within #12-DCV/ASV-pre.

Supplementary Materials. The NS3-to-NS5A long amplicon RT-PCR amplification.

Total RNA was extracted from 140 mL of serum using a QIAamp Viral RNA Mini kit (QIAGEN, Valencia, CA). HCV sequences spanning NS3 and NS5A regions (3120 bp) of the HCV genome for serum samples were reverse-transcribed in a volume of 20 μL with the One step RT-PCR Kit AMV or the PrimeScript One Step RT-PCR kit ver.2 (Takara Bio, Otsu, Japan) according to the manufacturer's protocol. For nested PCR, the products of RT-PCR were amplified using Phusion High-Fidelity DNA polymerase (FINZYMES, Espoo, Finland) or the PrimeStar HS kit (Takara Bio) according to the manufacturer's protocol by using HCV-specific primers shown in **Supplementary Table S8**.

Supplementary Tables.

Supplementary Table S1. The summary of SMRT sequencing result.

Supplementary Table S2. The error rate of HCV sequencing calculated from HCV-containing plasmids.

Supplementary Table S3. The codon information of 7 RASs.

Supplementary Table S4. All of the coded haplotypes in each sample.

Supplementary Table S5. Summary of the CCS2 reads including SVs.

Supplementary Table S6. List of structural variations existing at ≥1% in the sample.

Supplementary Table S7. Patients' information.

Supplementary Table S8. PCR primer set for sequencing the region between NS3 and NS5A genes.
